# A Framework for Studying the Heterogeneity of Risk Factors in Math Anxiety

**DOI:** 10.3389/fnbeh.2018.00291

**Published:** 2018-12-03

**Authors:** Orly Rubinsten, Hadas Marciano, Hili Eidlin Levy, Lital Daches Cohen

**Affiliations:** ^1^Edmond J. Safra Brain Research Center for the Study of Learning Disabilities, University of Haifa, Haifa, Israel; ^2^Department of Learning Disabilities, University of Haifa, Haifa, Israel; ^3^Department of Psychology, Tel Hai College, Kiryat-Shmona, Israel; ^4^Ergonomics and Human Factors Unit, University of Haifa, Haifa, Israel

**Keywords:** math anxiety, risk factors, framework, dynamic perspective, heterogeneity

## Abstract

Math anxiety is a prevalent disorder which affects many people worldwide. Here, we draw together ample evidence to suggest a dynamic developmental bio-psycho-social model. The model highlights the complex pathways towards the development of math anxiety, with a focus on dynamism. That is, math anxiety is viewed here as a dynamic interplay between environmental (parenting style, as well as social style including teachers’ attitude, instruction strategies and wider social effects) and intrinsic factors (i.e., neuro-cognitive and genetic predispositions, including brain malfunctions, heritability, predisposition towards general anxiety) and basic numerical cognition and affective factors. The model predicts that the dynamic interplay between these factors can either prevent or promote math anxiety’s effects on the development of heterogeneous symptoms. Considering the universal nature of math anxiety, a systematic description of the vulnerability factors that contribute to the development of math anxiety is vital. Such information may be of particular value in informing the design of preventive interventions as well as of specific intervention tools.

Math anxiety (Richardson and Suinn, 1972) comprises heterogeneous manifestations of symptoms (see Figure 1), including a negative attitude towards mathematics (Gierl and Bisanz, 1995; Dowker et al., 2012), feelings of stress in situations involving numerical information and avoiding activities which include numbers or quantities. High math anxiety levels are also associated with inflated physiological arousal during math related situations, such as taking a math test (e.g., Dreger and Aiken, 1957; Pletzer et al., 2010). Furthermore, math anxiety has consistently been shown to be negatively related to math achievement (e.g., Wigfield and Meece, 1988; Hembree, 1990; Ma, 1999). In the long run, math-anxious individuals are less likely to have math-related careers (science, technology, or engineering; see Hembree, 1990; Ma and Xu, 2004a, b; Ashcraft and Ridley, 2005; Maloney and Beilock, 2012). Math anxiety has been associated with increased health costs (Woloshin et al., 2001; Parsons and Bynner, 2005; Duncan et al., 2007; Reyna et al., 2009), low socioeconomic status (Ritchie and Bates, 2013) and mortgage default (Gerardi et al., 2013). In Western society poor numeracy is seen as a greater handicap than poor literacy (Rivera-Batiz, 1992; Estrada et al., 2004).

**Figure 1:**
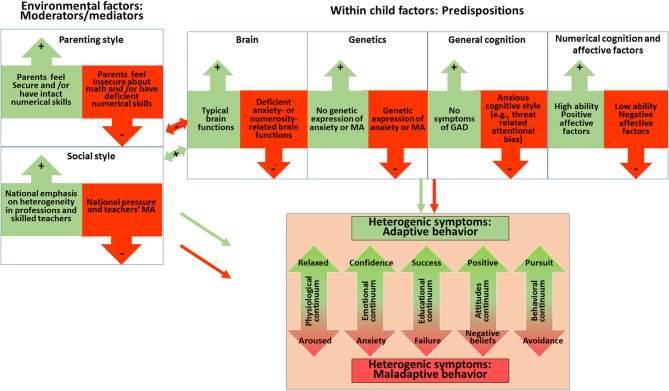
Math anxiety: a developmental dynamic bio-psycho-social model of math anxiety. The model predicts adaptive as well as maladaptive heterogeneity in behavioral outcomes (e.g., heterogeneous neurocognitive predispositions interact with parenting and social style during the child’s development). The model emphasizes two fundamental elements of the framework: 1. **Dynamic aspect**: the interactions between heterogeneous risk factors lead to math anxiety. A plus sign (+) within a green arrow means a beneficial interaction or influence, a minus sign (−) within a red arrow denotes an unfavorable interaction or influence; and 2. **Development aspect**: the effect of environmental factors (parental and social style) on development—these effects can act as both mediators or moderators (having both magnitude and direction towards the neurocognitive predisposition), or in some cases as an independent and direct developmental cause of math anxiety (e.g., when the child does not show any biological predispositions but the negative effects of the environment are severe, leading independently to math anxiety).

While understanding of the risk factors of math anxiety (Maloney and Beilock, 2012; Dowker et al., 2016; Suárez-Pellicioni et al., 2016) and its remediation (Supekar et al., 2015) has progressed, the question of why one person develops math anxiety while another does not, remains unresolved. Such understanding will enable new intervention programs to reduce the prevalence of math anxiety, which will address the range of possible antecedents of math anxiety rather than focusing solely on numerical skills (e.g., Supekar et al., 2015). Moreover, awareness of the entire spectrum of heterogeneous antecedents, may enable early identification of students who are at risk of developing math anxiety.

Indeed, a wealth of empirical evidence provides insights regarding distinct aspects of math anxiety (e.g., Daches Cohen and Rubinsten, 2017), including intervention studies on different features that are involved in its onset (e.g., Supekar et al., 2015) and systematic experimental studies with clinical populations (e.g., Lyons and Beilock, 2011) that target putative causal factors.

Here, we aim to assemble a range of data with relevance for issues of etiology, and to suggest a framework that organizes the entire spectrum of risk factors, for the study of math anxiety. This framework is concerned with the underlying mechanisms by which a variety of potential causes lead to math anxiety. The major assumptions of the current model are that mechanisms for math anxiety depend upon: (1) the interaction and dynamics between risk factors; and (2) changes in risk factors during human development. Thus, math anxiety is viewed here within the framework of a dynamic developmental perspective.

Although it is currently unfeasible to fully describe the origins and outcomes of math anxiety, we present an initial model focusing on the development and dynamic interplay between causal factors. Continuing previous important reviews of the antecedents of math anxiety and its developmental trajectory (e.g., Maloney and Beilock, 2012; Beilock and Maloney, 2015; Dowker et al., 2016), our current model highlights and visually illustrates dynamic and developmental perspectives claiming that heterogeneous symptoms of math anxiety result from the interplay between individual predispositions and one’s surroundings. The model predicts that the exact math anxiety symptoms at a particular time in life will be affected by factors that have positive or negative effects on the development of symptoms. The actual heterogeneous symptoms manifested are therefore a result of all of these different forces. Note that despite the importance of both antecedents and symptoms, and based on Sagvolden et al. (2005) the current article focuses on the heterogeneity and dynamics of the causes (i.e., risk factors) of math anxiety. Such a dynamic developmental model may open the door for future research to better understand the onset of math anxiety and to promote more comprehensive models.

## Developmental Perspective

There is currently insufficient evidence to determine whether the below mentioned factors (e.g., threat related attentional bias) are a cause or effect of math anxiety. However, longitudinal studies are beginning to examine these complex relationships. Recent studies have shown that the accumulation of negative experiences with learnt numerical information leads to math anxiety (Cargnelutti et al., 2017; Gunderson et al., 2018), emphasizing the importance of a developmental perspective. However, few studies have delineated developmental trajectories of the symptomatology of math anxiety and suggested moderate stability in the symptoms throughout middle childhood, especially for low math achievers. Specifically, a longitudinal study found that low scholastic achievers reported higher and stable math anxiety levels over time compared to high achievers who showed a higher anxiety level at the beginning and the end of the school year, which after an adjustment period reverted to its lower level, suggesting susceptibility to changes over time (Madjar et al., 2016). Indeed, there are known accounts of math anxious people during childhood who later overcame their anxiety to become proficient mathematicians and even Fields Medal winners (considered the mathematician’s “Nobel Prize”), such as Prof. Laurent Schwartz who wrote in his autobiography: “‥‥towards the end of the eleventh grade, I secretly thought of myself as stupid. I worried about this for a long time” (Schwartz, 2001). Similarly, Prof. Maryam Mirzakhani, the first woman to win the Fields Medal, had a middle school math teacher who thought that she was not talented, which undermined Mirzakhani’s confidence^1^.

Thus, our model suggests heterogeneity in the continuity and manifestations of the math anxiety disorder, with expressions of math anxiety symptoms varying at different developmental phases, due to the dynamic interplay between environmental and intrinsic factors.

## The Dynamic Perspective and a General Description of the Model

The model comprises a dynamic bio-psycho-social perspective describing how individual variations in competing factors of neuro-cognitive and genetic predispositions (*within-child*), together with parenting style and social style* (environmental factors)*, may affect math learning processes and emotional functions, thereby producing heterogeneity within math anxiety (Figure 1). These factors can either interact or cancel each other during development. Environmental factors refer to the feelings and actions of parents, teachers and other formal agents of society, regarding mathematics. While some of them feel secure about their own math abilities and transfer their secure feelings to the child, others suffer themselves from math anxiety or math difficulties and may project these maladaptive patterns to the child (Beilock et al., 2010). Environmental risk factors for math anxiety can be found in school (Beilock et al., 2010; Gunderson et al., 2012), during extra-curricular activities (Berkowitz et al., 2015), and within the child’s family (Berkowitz et al., 2015; Maloney et al., 2015; Daches Cohen and Rubinsten, 2017; for protective parental effects see Gunderson et al., 2018).

The* within-child* factor refers to the child’s predispositions, related either to *neural correlates*, the child’s* genetics*, the child’s tendency toward *anxiety in general* (Generalized Anxiety Disorder, GAD), or *basic numerical skills and affective factors*. Specific neural correlates can involve, for example, mathematics-related brain dysfunction in the intraparietal sulcus, that increase the probability of exhibiting math anxiety (Young et al., 2012). *Genetic heritage* refers to the genetic root of math anxiety, found mostly through studies of twins (Wang et al., 2014; Malanchini et al., 2017). The question regarding *basic numerical cognition and affective factors* seeks to determine whether deficient numerical skills lead to math anxiety (e.g., Ma and Xu, 2004a) or rather math anxiety leads to deficient mathematical performance (e.g., Ashcraft and Kirk, 2001; Maloney and Beilock, 2012; Park et al., 2014). The *tendency toward GAD* suggests that children predisposed to GAD (Hembree, 1990; Ashcraft and Moore, 2009; O’Leary et al., 2017), including maladaptive threat related attentional bias (excessive attention to threat stimuli, Bar-Haim et al., 2007; Bishop, 2009; Cisler and Koster, 2010; Luijten et al., 2012), are at greater risk of developing math anxiety. All of these within-child factors affect how the individual interprets communication signals from the environment (e.g., Chorpita et al., 1996; Dineen and Hadwin, 2004; Affrunti and Ginsburg, 2012).

The model indicates that even if one or more of these predispositions exists, math anxiety will not necessarily develop. Other environmental factors, serving as mediator or moderator variables, including parenting and/or social style, may help the child “overcome” the deficits, generating adaptive behavior.

Importantly, the link between environmental and within-child factors (pre-dispositions) is bidirectional. This perspective expands other models that aim to identify the factors that individually and collectively account for large amounts of variance in math and science achievement, such as the Opportunity-Propensity (O-P) Model (Byrnes and Miller, 2007; Byrnes and Wasik, 2009; Baten and Desoete, 2018). The O-P model assumes that distal factors (e.g., socioeconomic status) predict the proximal factors, which include opportunity factors (e.g., coursework) and propensity factors (e.g., prerequisite skills). These proximal factors, in turn, predict the developmental outcomes.

In contrast, in the current model children are not passively influenced by their teachers, parents and the larger culture. Rather, we emphasize a bi-directional link in which environmental factors can be influenced by characteristics of the student, which in turn may influence the student’s math anxiety. Indeed, research shows that individuals can be dynamic producers of their environment (e.g., Lerner, 2001; Heckhausen et al., 2010; Lerner and Busch-Rossnagel, 2013). Namely, individuals can actively change their environment by, for example, extracting different responses from others, or by understanding and interpreting communications in distinctive ways (Dineen and Hadwin, 2004).

Hence, as can be seen in Figure 1 and as elaborated below, environmental factors can act as moderators that reduce the effect of pre-dispositions on the development of math anxiety, but they can also act as mediators and affect the pre-dispositions themselves. Similarly, either innate (i.e., pre-disposition) or learnt (environment) numerical skills are suggested to have a reciprocal relationship with math anxiety.

Note that the *within-child* factors are seen as predispositions for MA, while environmental factors may act as either mediators or moderators. Therefore, in what follows, they will be described first. However, it should be stressed that these predispositions are not mandatory or necessary in order to develop math anxiety, because the other (environmental) factors, either alone or together, may act as an independent direct developmental cause of math anxiety. Thus, the actual severity and specific symptoms of math anxiety (see the bottom of Figure 1) are heterogeneous and depend on the dynamic pattern between all of the following risk factors.

## Within-Child Factors: Biological Predispositions

### Genetic Predisposition

*Genetic* studies may reveal whether genetic predisposition does in fact emerge first to cause math anxiety development. A recent study on monozygotic vs. same-sex dizygotic teenage twins found that genetic factors accounted for about 40% of the math anxiety variance and that 9% of the total variance in math anxiety resulted from genes related to GAD (Wang et al., 2014). Similar results were found with older twins (Malanchini et al., 2017), suggesting that some of the origins of math anxiety rely on genetic factors. According to Dowker et al. (2016), it is reasonable to assume that no genetics factors are specific to math anxiety. Indeed, in a multivariate analysis (Wang et al., 2014), math anxiety was influenced by the genetic and nonfamilial environmental risk factors involved in general anxiety and the genetic factors were associated with math-based problem solving. Therefore, math anxiety may result from a combination of negative experiences with math and genetic predisposition associated with both math cognition and general anxiety.

### Neural Predisposition

Neuronal correlates of math anxiety have been linked to different brain activation patterns (Artemenko et al., 2015), which relate to affective or cognitive mechanisms. In one study (Young et al., 2012) children with math anxiety exhibited reduced activity in the posterior parietal and dorsolateral prefrontal cortex (DLPFC) regions (known to be involved in mathematical reasoning), on the one hand, and higher and abnormal effective connectivity between the amygdala and ventromedial PFC regions that regulate negative emotions, such as fear, on the other. Specifically, among the high math-anxious children, the amygdala showed greater coupling with cortical regions involved in processing and regulating negative emotions, whereas in their low math-anxious peers, the amygdala was coupled with brain areas that facilitate efficient task processing.

Others (Lyons and Beilock, 2012) found increased activation in the pain perception network, including the bilateral dorso-posterior insula and mid-cingulate cortex, among high math-anxious individuals who anticipated a math task. Interestingly, these areas showed no significant activation during completion of the task. It seems that before engaging in math tasks, math anxious individuals show pain-related activity, while during the actual math engagement they show fear-related neural activity (Artemenko et al., 2015). Similarly, high math-anxious individuals demonstrated greater involvement of the right insula for numeric than for nonnumeric errors (Suárez-Pellicioni et al., 2013a), a brain structure that has been associated with pain (Isnard et al., 2011), emotional processing (Phan et al., 2002) and anxiety disorders (Paulus and Stein, 2006). These results may explain the avoidance behavior that characterizes math-anxious individuals (Hembree, 1990; Ashcraft and Ridley, 2005).

Importantly, while anticipating a math task, increased activation in the frontoparietal network, which is known to be involved in regulating negative emotions (including the inferior frontal junction, the inferior parietal lobule and the left inferior frontal gyrus), predicted activation of the right caudate nucleus and the left hippocampus when performing a math task among high math-anxious individuals. These two subcortical regions are necessary for coordinating task demands and motivational factors (Lyons and Beilock, 2012). Thus, activation of brain substrates of the cognitive control function when anticipating a math task can mitigate the effects of math anxiety on performance. In this line of research, high math-anxious individuals demonstrated reduced activity in the DLPFC, a brain region which is also thought to be involved in cognitive control (Young et al., 2012). Moreover, transcranial direct current stimulation (tDCS) to the DLPFC improves the performance of high math-anxious individuals and even ameliorates their anxious response, as reflected in declines in cortisol concentrations (Sarkar et al., 2014).

Nevertheless, it is not clear whether these neural patterns are a cause (predisposition) or effect of math anxiety, a question that remains to be resolved by future longitudinal studies (e.g., Luo et al., 2011). Furthermore, the reported brain activation patterns can also reflect individual differences, as well as cognitive strategy differences. For example, increased math anxiety levels were associated with low math achievements, as well as with reduction of gray matter volume in the left anterior intraparietal sulcus, a region associated with attention processing (Hartwright et al., 2018). Concurrent with the attentional control theory (Eysenck et al., 2007), the authors hypothesized that both increased math anxiety and limited attentional resources may reduce math achievements by depleting working memory capacity.

### Cognitive and Neuro-Physiological Predisposition: The Link With Generalized Anxiety Disorder (GAD)

The neural component of math anxiety is further demonstrated in studies showing a correlation between GAD and math anxiety (Hembree, 1990; Wang et al., 2014; Malanchini et al., 2017). Our model claims that sometimes math anxiety is linked, at least during initial development, to symptoms of GAD. Intrinsically vulnerable people tend to hold cognitive traits of anxiety (e.g., maladaptive threat-related attentional bias), as well as maladaptive neuro-physiological responses, which can constitute an independent risk factor for math anxiety. Indeed, general-anxiety related **neuro-physiological responses** and increased negative emotional processing have been found in young children with math anxiety, as indicated by hyperactivity in the right amygdala during math performance (e.g., Cargnelutti et al., 2017). Currently, there is insufficient evidence to conclude whether GAD acts as a predisposition in cases of math anxiety. However, longitudinal studies are beginning to examine these relationships. For example, in a longitudinal path model, GAD was found to be a significant predictor of math achievements, stronger in second than in third grade (Cargnelutti et al., 2017).

A leading theory explaining **the cognitive aspects** of math anxiety suggests that individuals with math anxiety do not necessarily suffer from math learning disabilities (Ashcraft and Kirk, 2001; Ashcraft et al., 2007). Rather, they suffer from typical GAD symptoms, specifically math anxiety-induced ruminations (repetitive thinking about negative personal concerns, Nolen-Hoeksema et al., 2008). These ruminations jeopardize cognitive resources such as working memory. As a consequence, insufficient cognitive resources lead to lower performance on numerical tasks (Beilock, 2010; Maloney and Beilock, 2012; Ramirez et al., 2016). However, note that others have suggested a bidirectional link between math anxiety and math performance, meaning that math anxiety can be simultaneously both the cause and consequence of math difficulties (Carey et al., 2016), as will be detailed below.

Against this background, we argue that some of the cognitive traits associated with GAD, such as attentional bias toward negative information (Muris et al., 2003; Ashcraft and Moore, 2009) which leads to anxiety-induced ruminations, are also involved in math anxiety. Though selective attention to threat reflects the adaptive neurocognitive function of protection from danger (Bar-Haim et al., 2007, 2010; Robinson et al., 2012), dysfunction in this process results in threat-related attentional bias (Bar-Haim et al., 2007, 2010; Ashcraft and Moore, 2009; Bishop, 2009; Van Bockstaele et al., 2014; O’Leary et al., 2017). Indeed, we and others (Rubinsten et al., 2015; Suárez-Pellicioni et al., 2015) have shown maladaptive threat-related attentional bias towards basic numerical information in adults with math anxiety. This finding suggests that math anxiety results from maladaptive characteristics of GAD that lead to experiencing the world, and mainly the parts associated during development with negative valence (e.g., numerical information), as threatening. Attentional bias toward threat-related stimuli, in turn, interfere with attentional control and increase sensitivity to distractions (Suárez-Pellicioni et al., 2013b, 2014) or to the inhibition of anxiety-related responses (Pletzer et al., 2015). Therefore, if a child with GAD encounters adverse numerically-related pedagogical or social events, the risk of developing math anxiety increases.

### Numerical Cognition and Affective Factors

Math anxiety has consistently been shown to be negatively related to math achievement (e.g., Wigfield and Meece, 1988; Hembree, 1990; Ma, 1999). As an example, math anxiety have been found to be associated with basic numerical skills, such as simple counting (Maloney et al., 2010; Rubinsten and Tannock, 2010; Rubinsten et al., 2012; Núñez-Peña and Suárez-Pellicioni, 2014). In addition, children with diagnosed mathematical disabilities exhibited more math anxiety compared to a control group (Rubinsten and Tannock, 2010; Passolunghi, 2011). Furthermore, it has been shown that math anxiety and math ability share common genetic etiology (Wang et al., 2014).

Nevertheless, the question of whether deficient numerical skills lead to math anxiety or rather math anxiety leads to deficient mathematical performance, is still under debate. Recent findings cast doubt on the idea that deficits in basic numerical processing underlie math anxiety (e.g., Maloney et al., 2010, 2011). Devine et al. (2018) found a dissociation between cognitive and emotional math problems in a large sample of children, despite having a significant percentage of co-occurrence. In addition, the assumption that math anxiety influences numerical cognition skills is supported by the finding showing that children diagnosed with math learning disabilities in grades 4–7 demonstrated an increase in math performance after training that helped them reduce their math anxiety level (Kamann and Wong, 1993). It is suggested that worries and intrusive thoughts characteristic of anxiety, may disrupt thinking processes when faced with a math task (Chang and Beilock, 2016) and thus, consume valuable attentional resources (Ashcraft and Kirk, 2001; Maloney and Beilock, 2012; Park et al., 2014). However, note that longitudinal studies suggest that math anxiety may be the result of a deficit in numerical cognition skills in students who began school as low mathematics achievers (Ma and Xu, 2004a).

Recent research implies that the relationship between math anxiety and math performance is bidirectional (Carey et al., 2016; Foley et al., 2017; Ramirez et al., 2018). For instance, a longitudinal study involving very young students (first to second grade) indicates the although math performance level at school entry (Duncan et al., 2007) is a strong predictor of math anxiety, a reciprocal relationship between math anxiety and math performance was observed even after a short exposure to formal education (Gunderson et al., 2018). Others have shown that there may be unidirectional relations between math anxiety and poor math performance (e.g., Ashcraft and Moore, 2009). Math anxiety can have a direct effect on math performance (Ashcraft and Kirk, 2001; Beilock and Maloney, 2015), but also an indirect effect through avoidance behavior (Hembree, 1990; Ashcraft and Ridley, 2005).

It may be concluded, then, that in some cases deficient numerical skills can result in math anxiety, but depending on the interaction with other risk factors (e.g., Figure 1), math anxiety may harm mathematical performance.

In addition to numerical cognitive factors, affective factors, such as motivation or self-esteem, may serve as protectors. For example, math motivation can help overcome anxiety-related responses (Chang and Beilock, 2016). In support of this notion, a negative linear correlation has been observed between math anxiety and math performance in adolescents and adults with low math motivation, whereas an inverted-U curvilinear relationship has been found in more motivated students (Wang et al., 2015). Accordingly, contemporary functional MRI research has indicated that high activations in motivation-related brain regions, reduces the negative effects of high math anxiety on performance (Lyons and Beilock, 2011).

Similarly, thoughts and feelings about the self have been found to have an important role in educational outcomes (Lee, 2009). For example, Hembree (1990) found a negative correlation between math anxiety and enjoyment of math, self confidence in math, opinion about the usefulness of math and attitudes toward math teachers. In Hoffman’s (2010) study, the compensatory relationship between self-efficacy and math anxiety was related to problem-solving efficiency. This finding supports the role of self-efficacy, which is defined as one’s self-belief about the ability to produce successful outcomes (Bandura, 1997), in reducing math anxiety (Lee, 2009). Likewise, self-concept, which refers to how the individual perceives his or her own ability in a specific discipline (Bandura, 1986; Marsh, 2006), has also been found to mediate the link between math anxiety and performance among third and fifth graders (Justicia-Galiano et al., 2017). However, other researchers have argued that low self-concept and self-esteem is the result of math anxiety (Ahmed et al., 2012). Recently, Mammarella et al. (2018) demonstrated that the lower the self-concept, the higher the risk of developing anxiety. Similarly, path analysis and cluster analysis showed that academic resilience is predicted by self-efficacy (confidence), coordination (planning), control, commitment (persistence) and highly relevant to the current model, low anxiety (Martin and Marsh, 2006). In conclusion, current research is inconclusive and suggests that affective factors may act either as mediating or moderating math anxiety or even as part of the heterogeneous symptoms.

### Summary: Predispositions and Clinical Interventions

Within-child factors may serve as predispositions for math anxiety, but the opposite is also true. As indicated in Figure 1 and in the above summary, people who acquire none of these predispositions should be less prone to develop math anxiety. Regardless of the specific within-child factor, math anxiety narrows the working memory span when dealing with math tasks (Ashcraft and Kirk, 2001; Passolunghi et al., 2016; Shi and Liu, 2016; Ching, 2017; for working memory and anxiety see Moran, 2016). Therefore, suggested interventions for math anxiety typically include (general) anxiety-reducing methods. Examples of such interventions include Acceptance and Commitment Therapy (ACT) or systematic desensitization (Zettle, 2003), focused breathing exercise prior to the arithmetic test (Brunyé et al., 2013), emotion regulation strategies (Pizzie and Kraemer, 2018) and extensive one-on-one math tutoring (Supekar et al., 2015). It has been shown that reducing typical anxiety symptoms frees more working memory resources for math performance. Yet, the improvement in math performance after math tutoring (Supekar et al., 2015) can also account for the reduction in math anxiety levels by reducing failure experiences.

## Environmental Factors: Mediating and Moderating Processes

Within-child factors provide only a partial explanation of math anxiety development. In the above cited twin study, for example, a major proportion of the variance in math anxiety was accounted for by non-shared environmental factors (Wang et al., 2014).

Children spend most of their life at home or school, and are strongly influenced by interactions with parents, family members, teachers and peers. Our model suggests that a history of mathematically-related aversive outcomes resulting from social interactions, as well as poor modeling (e.g., teachers with math anxiety) or inappropriate math instruction strategies, may all lead to negative beliefs about mathematical competence and contribute to the development of math anxiety (Beilock et al., 2010). Indeed, numerous studies show that inappropriate instruction strategy may lead to the development of math anxiety (e.g., Baroody and Hume, 1991; Jackson and Leffingwell, 1999). According to Curtain-Phillips (1999), teachers need to re-examine traditional teaching methods and use methods which include less lectures, more student directed classes and more discussion. Thus, adverse mathematically-related pedagogical and social events during development, particularly those involving teachers (Beilock et al., 2010; Gunderson et al., 2012) and parents (Gunderson et al., 2012; Maloney et al., 2015; Daches Cohen and Rubinsten, 2017), are dynamically combined with maladaptive predispositions and may lead to math anxiety. We elaborate below.

### Parenting Style

Though many parents see math education as the school’s responsibility (Cannon and Ginsburg, 2008), children themselves seek math help from their parents and teachers expect parents to help their children with their homework (Maloney et al., 2015). However, parenting practices, such as pressure to maintain high achievements (Daches Cohen and Rubinsten, 2017) and involvement in math-learning processes (Roberts and Vukovic, 2011), have been found to increase children’s math anxiety. In particular, math-anxious parents may trigger, or intensify, their child’s math anxiety, especially when these parents report being extensively involved in helping their child with math homework (Maloney et al., 2015; Daches Cohen and Rubinsten, 2017).

The effect of parental math anxiety on children’s math anxiety level was mostly demonstrated by studying mother-child dyads (Casad et al., 2015; Maloney et al., 2015; Daches Cohen and Rubinsten, 2017), but in at least two studies (Casad et al., 2015; Maloney et al., 2015) the sample was not initially limited to mothers. Additionally, one study found that mother-daughter dyads showed the most significant parental math anxiety effect (Daches Cohen and Rubinsten, 2017). A longitudinal study confirmed the effect: that is, the perceptions of mothers regarding their 7th grade children were associated with the career choices of their daughters, but not of their sons (Casad et al., 2015). Such gender bias patterns can explain why more males take advanced math courses and choose math related careers (Bleeker and Jacobs, 2004; Gunderson et al., 2012).

Parents, especially mothers (who were simply those that were studied the most), have an important role in the belief systems of their children, especially daughters, regarding math ability, self-efficacy and math anxiety. Thus, it may be important to encourage parents to act in order to reduce their own math anxiety as well as that of their children, and to encourage their children to pursue challenging courses and careers (Scarpello, 2007).

### Social Style: Teachers

Math anxious teachers may generate math anxiety in their students and affect its intensity (Martinez, 1987). However, this pattern seems to exhibit gender asymmetry. The level of math anxiety among elementary school female teachers was found to affect the math achievements of their female but not of their male students (Beilock et al., 2010). Similarly, female students of high math-anxious female teachers acquired negative stereotypes about girls and math, known to be related to math anxiety (Hembree, 1990). The mechanism of this gender asymmetry might be explained by the identification process of girls with their female teachers as a same-gender role model (Bussey and Bandura, 1984), which probably pushes girls to identify with their female teacher’s negative attitude toward math (Gunderson et al., 2012).

#### Teachers and Learnt Numerical Information

Math anxiety was also found to have a strong link with the learnt symbolic numerical information (such as arithmetic or Arabic numerals; e.g., Maloney et al., 2011; Dietrich et al., 2015). This link reflects the complex pathways towards the development of math anxiety, since it has several possible explanations, such as a genetic risk factor associated with poor math ability, an underlying neurological deficit in symbolic numerical processing, or exposure to inappropriate math instruction strategies. Negative pedagogical experiences with these learnt symbols (Cargnelutti et al., 2017; Gunderson et al., 2018) may lead to threat related attentional bias specifically towards learnt numerical information (that has been linked to negative valence during development, see Rubinsten et al., 2015). Supporting the argument that math anxiety is activated specifically during involvement with learnt numerical information, math-anxious individuals were found to exhibit a deficit in the counting (an exact symbolic linguistic skill strongly associated with formal schooling; Gallistel and Gelman, 1992; Cordes et al., 2001) but not in the subitizing range (an exact symbolic linguistic skill that usually develops in the preschool years), with working memory as a mediator of this effect (Maloney et al., 2010). Moreover, math-anxious individuals showed reduced accuracy in mental representations of learnt symbolic but not innate non-symbolic numerical magnitudes or innate spatial abilities (Maloney et al., 2011; Dietrich et al., 2015; Douglas and LeFevre, 2018).

#### Teachers, Parents and Negative Beliefs

We propose that an accumulation of adverse pedagogical and social events during development, involving teachers (Beilock et al., 2010; Gunderson et al., 2012) and parents (Gunderson et al., 2012; Daches Cohen and Rubinsten, 2017; Maloney et al., 2015), in *dynamic* combination with maladaptive mechanisms (predispositions), may lead to math anxiety through the creation of negative beliefs acting either as a result of math anxiety (symptoms; see bottom of Figure 1) or as mediators, moderators, or causes of math anxiety. Research into the mediating pathways between adverse environmental experiences and negative beliefs is in its infancy, but a recent study found that the more teachers emphasized the need to demonstrate competence in the classroom, the more their students believed that ability is stable and inflexible (Park et al., 2016). Similarly, it has been shown that parents and teachers significantly shape the child’s math attitudes (Gunderson et al., 2012; Park et al., 2016). Future longitudinal studies can help reveal the actual causal direction.

### Wider Social Effects

Cultural norms have a significant effect on math anxiety expression (Stoet et al., 2016; Foley et al., 2017). For example, countries with higher math achievement have fewer students with math anxiety and vice versa (Organization for Economic Co-operation and Development, 2013). Additionally, Asian countries (Korea, Japan and Thailand) showed a high prevalence of math anxiety, while Western European countries (Austria, Germany, Liechtenstein, Sweden and Switzerland) showed a low prevalence of math anxiety (Lee, 2009). Moreover, students in some high math achieving countries (mostly East Asian, such as Singapore or Korea) showed a high prevalence of math anxiety, while a low prevalence of math anxiety was evident among students in other high achieving countries (such as Switzerland; Foley et al., 2017). This disparity can be attribute to cultural differences, due to the pursuit of high academic achievements across high math achieving countries, inducing high math anxiety among students in these countries (Stankov, 2010). Though the symptoms of math anxiety are relatively constant across cultures, their expression varies depending on the mathematical concerns of a given culture. For example, gender differences in math anxiety may partly result from cultural pressure to reduce math anxiety in males while tolerating it in females. Indeed, economically-developed and gender-equal countries showed lower overall math anxiety occurrence, but a surprisingly larger national gender difference in the distribution of math anxiety (Stoet et al., 2016) which may be attributed, in part, to the well-established gender differences in occupational interests (Leder, 1990; Kenway and Willis, 1993; Su et al., 2009). Finally, culture may influence the point at which math anxiety is seen as an obstacle in one’s life, thus affecting diagnosis rates. Given that most individuals spend their entire life in a given culture, its characteristics may be important for manifestations of math anxiety across the lifespan.

### Summary: Environmental Factors and Clinical Interventions

Countermeasures for these environmental factors must stem from the education curriculum and cultural/social climate change (Martinez, 1987; Scarpello, 2007; Organization for Economic Co-operation and Development, 2013). For example, math anxiety was reduced when using teaching methods that concretize mathematics concepts (Vinson, 2001). Martinez (1987) listed several guidelines for an anxiety-free class and suggested that interventions that focus on the teacher’s own math fears might “break the chain” and prevent the continuous spreading of math anxiety. With a wider cultural perspective, the education system should be more sensitive to the different needs of males and females, as their attitudes toward math may differ (Geist, 2010).

## Conclusion

The current model focuses on the trajectories of math anxiety from a developmental, dynamic and bio-psycho-social perspective, which hold that math anxiety results from multiple causal interacting influences. Namely, heterogeneous symptoms of math anxiety emerge from multiple developmental pathways that reflect the dynamic interplay between characteristics of children (intrinsic predispositions including heritage, neural functions and cognitive-processing) and their environment (teachers, parents and wider social effects) over time. Although some of the relationships are still hypothetical, the evidence to support such a model is accumulating and indicates that predispositions and environmental factors may indeed enhance or counteract each other.

Thus, for example, for intrinsically biologically vulnerable individuals, adverse math learning experiences may result in threat-related attentional bias towards learnt numerical information as well as in maladaptive beliefs about individual mathematical (dis)abilities, with difficulties in controlling the outcomes of mathematical situations, which may lead to ruminations. In turn, these negative beliefs and ruminations may be linked to behavioral, somatic and emotional responses of math anxiety (see heterogeneous symptoms—bottom of Figure 1). Avoidance of future numerical activities may also act as a behavioral manifestation of math anxiety (Ashcraft, 2002), which in turn reduces positive opportunities to acquire numerical skills and limits the development of good math proficiency (Krinzinger et al., 2009). Thus, a vicious cycle might be established, increasing the math anxiety level.

Future research should study the developmental course of math anxiety, in an attempt to identify different groups of individuals with distinct trajectories, critical to understanding the heterogeneity of math anxiety symptoms. In addition, future studies are urged to explore the additive or interacting effects of multiple factors in the development of math anxiety.

From a practical point of view, intervention programs should take into account the complex pathways towards the development of math anxiety, which lead to heterogeneity in the continuity and manifestations of math anxiety, beyond focusing on (general) anxiety-reducing methods. Awareness of the heterogeneity in both causes and symptoms of math anxiety will enable educators and psychologists to identify as early and accurately as possible students who have developed or are at risk to develop math anxiety. Early and accurate diagnosis of math anxiety will, in turn, allow implementation of a Response to Intervention (RTI) model which has three basic components (Fuchs and Fuchs, 2005; Brown-Chidsey and Steege, 2011). First, choosing a research-based intervention that matches the student’s educational and behavioral needs. Second, using progress monitoring in order to consider the need for changes in instruction or in the goals of intervention. The third component includes making educational decisions about the extent of support provided to students according to their therapeutic history and the information obtained in the progress of monitoring the severity of their difficulties.

## Author Contributions

OR conceived of the presented idea and developed the theory. All authors discussed the theory and contributed to the final manuscript. All authors participated in the writing and the revision of the manuscript.

## Conflict of Interest Statement

The authors declare that the research was conducted in the absence of any commercial or financial relationships that could be construed as a potential conflict of interest.

## Footnotes

^1^Taken from https://www.wired.com/2014/08/maryam-mirzakhani-fields-medal/
